# Development of a New, High Sensitivity 2000 kg Mechanical Balance

**DOI:** 10.3390/s17040851

**Published:** 2017-04-13

**Authors:** Jian Wang

**Affiliations:** Division of Mechanics and Acoustics, National Institute of Metrology, Beijing 100029, China; wjian@nim.ac.cn; Tel.: +86-10-6452-4609

**Keywords:** sensitivity, mechanical balance, laser displacement sensor, repeatability, synchronous

## Abstract

Mass measurement of more than 500 kg on an electronic mass comparator has no better repeatability and linearity of measurement for meeting the calibration requirement of over class F_1_ weights from pharmacy and power generation plants. For this purpose, a new 2000 kg mechanical balance was developed by the National Institute of Metrology (NIM). The advantages of measurement of more than 500 kg on a new 2000 kg mechanical balance are introduced in the paper. In order to obtain high measurement uncertainty, four vertical forces of two sides of beam are measured and used as reference for adjustment of the beam position. Laser displacement sensors in the indication system are more effective for decreasing reading errors caused by human vision. To improve the repeatability and sensitivity of the equipment, a synchronous lifting control is designed for synchronously lifting the beam ends along the vertical direction. A counterweight selection system is developed to get any combination of weights in a limited space. The sensitivity of the new mechanical balance for 2000 kg is more than 1.7 parts in 10^−4^ rad/g. The extended uncertainties for the mechanical balance of 500 kg, 1000 kg and 2000 kg are 0.47 g, 1.8 g and 3.5 g respectively.

## 1. Introduction

The calibration requirement of high accuracy for big weights or loads like 500, 600 and 1000 kg is increasing year by year in China. Electronic mass comparators are usually used for calibrating high accuracy weights all over the world. The repeatability and linearity of electronic mass comparators are good for meeting the calibration requirement of over class F_1_ weights, except for mass comparators with a maximum capacity of more than 500 kg [1,2,3].

The repeatability and linearity of a mass comparator depends on its lever structure, the position of the applied gravity force and the performance of its load cells. The lever structure in a mass comparator, consisting of a pivot and metal beam, is used to amplify the force of gravity during the mass measurement. The material and deformation of the metal beam are the factors that influence and amplify the accuracy. The precision of the amplification on a small mass comparator is better than that on a large mass comparator. For large electronic mass comparators based on lever structures it is difficult to get better repeatability and linearity in the mass measurement process.

A weight placed on different position of the platform could result in a difference between two adjacent measurements. This is more obvious for big weights over 100 kg in mass. For obtaining better measurement result repeatability, the bottom position of the weight on the platform is usually marked. This ensures the weight will be placed on the same position each time during the measurement process.

Due to the characteristics of load cells, electronic mass comparators with different maximum capacities may be selected when used for measuring the masses of weights with the same accuracy and different nominal values. It is necessary for the weights mentioned above to use several electronic mass comparators for performing the corresponding measurements.

The mechanical balance with one indicator based on the optical principle is commonly used all over the world. Usually the operator participates in the data acquisition, and automatic data acquisition is not available. This method introduces some reading uncertainty. It causes an increase in the measurement uncertainty of a mechanical balance [4,5]. A new method based on laser displacement sensors is proposed to resolve the problem in this paper.

Due to the different weight of the measured weight, the counterweights are usually not installed on the mechanical balance. When used, counterweights are selected to be loaded on the side of the mechanical balance [6]. This is not convenient to operate and it affects the measurement accuracy. To resolve this problem, a special design with counterweights mounted on the mechanical balance is introduced in this paper.

The beam of a mechanical balance has better stiffness and less deformation than that of an electronic mass comparator. Based on this characteristic, we have developed a new mechanical balance with a maximum capacity of 2000 kg. The sensitivity of the mechanical balance for 2000 kg is more than 1.7 parts in 10^−4^ rad/g. The repeatability for 2000 kg is less than 0.15 g. It could measure weights with different nominal values from 100 kg to 2000 kg.

## 2. Structure of Mechanical Balance

**Figure 1:**
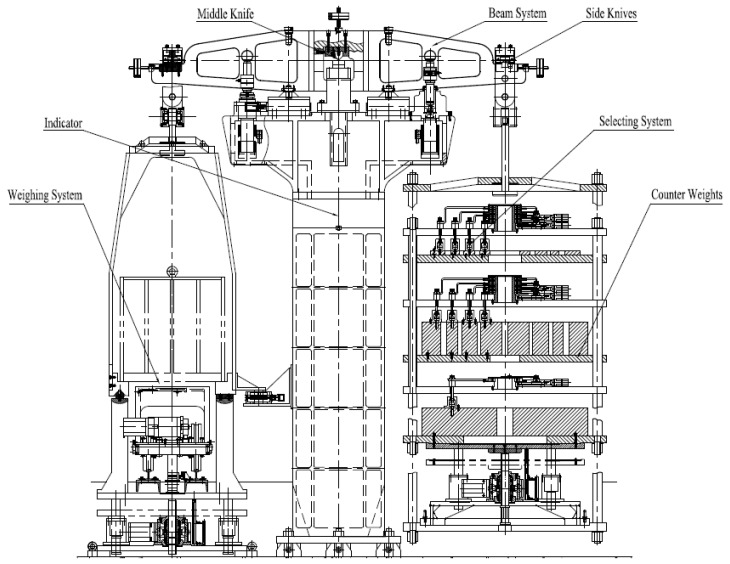
The structure of the mechanical balance.

The mechanical balance is composed of beam system, middle knife, side knives, indicator, counterweights, selecting system, weighing system, motors and controller. The structure of the mechanical balance is shown in Figure 1.

**Figure 2:**
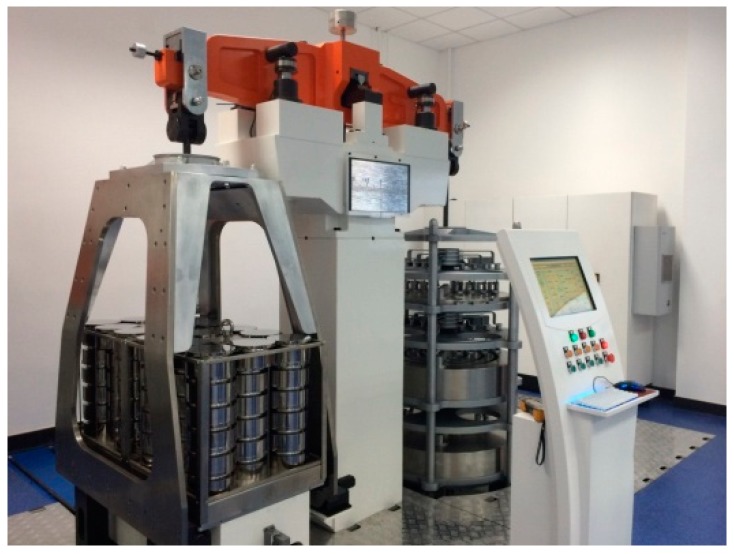
The picture of mechanical balance.

Figure 2 is a picture of mechanical balance. The height of the mechanical balance is 2.8 m. The length of the balance beam equals 2 m.

### 2.1. Knives of Mechanical Balance

**Figure 3:**
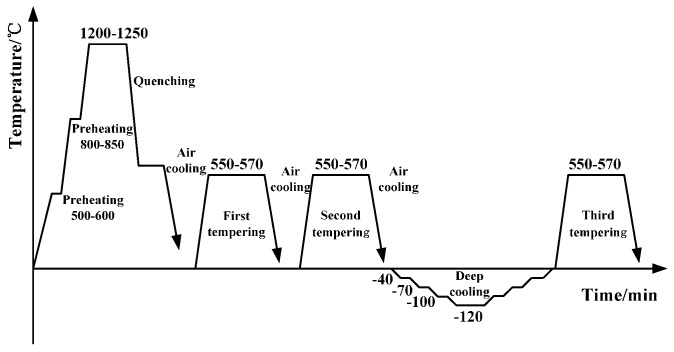
Heat treatment process of TH10.

For supporting an approximate 7 t load, TH10 high-speed steel is used as the material of the middle knife and side knives after a special heat treatment process as shown in Figure 3. The special heat treatment process includes preheating two times at 500–600 ℃ and 800–850 ℃, respectively, quenching at 1200–1250 ℃, tempering three times at 550–570 ℃ and a deep cooling cycle down to –120 ℃ [7]. After machining the knives have maintained high hardness and high toughness under heavy load conditions [8].

### 2.2. Indicators of Mechanical Balance

**Figure 4:**
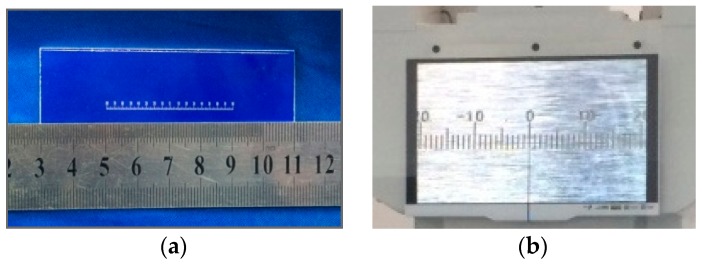
(**a**) The scale and (**b**) the LCD.

The display device includes two individual indicators. One is an indicator based on optical principles. It is composed of a small scale, a high definition camera and a liquid crystal display (LCD). The scale and the LCD are shown in Figure 4. A metal thin rod fixed in the middle of beam is perpendicular to the beam. The scale of the indicator is mounted on the end of the metal thin rod and parallel to the beam. The distance between the middle knife-edge and the scale is 42 cm. The scale swings with the swing of the beam and includes 160 divisions. The interval between divisions of the scale is equal to 0.25 mm. A high definition camera with 14 million pixels is used for amplifying the scale and displaying it on the LCD. During the measurement, the operator reads the LCD readings.

**Figure 5:**
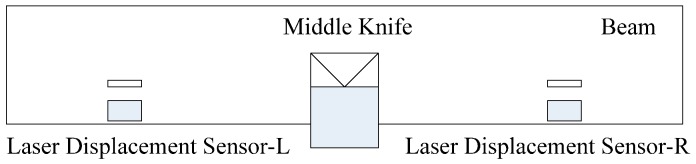
The installation of two laser displacement sensors.

Another includes two laser displacement sensors, a signal processor and a computer. Laser displacement sensors with the resolution of 3 μm are mounted on both sides of the beam. The distance between the middle knife-edge and each laser displacement sensor is 42 cm. The installation of two laser displacement sensors is shown as Figure 5. When the beam swings, the laser displacement sensor-L gets the displacement corresponding to equilibrium position on the left side. Meanwhile the laser displacement sensor-R gets the displacement on the right side. The difference of displacements of both sides is the displacement of the beam. It can eliminate the influence of the zero drift of the laser displacement sensor. The difference is processed by the signal processor and sent to the computer.

**Figure 6:**
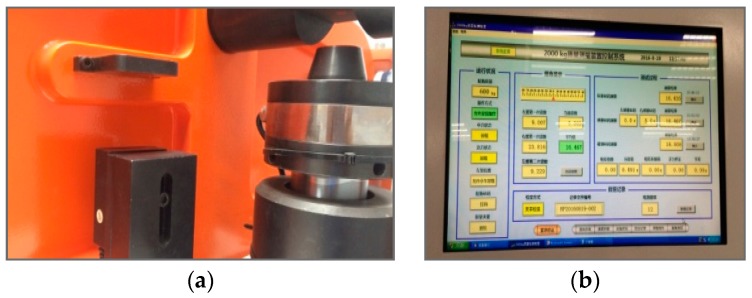
(**a**) The position of one sensor and (**b**) the software interface.

The indicator based on optical principle is used for adjusting the indicator with two laser displacement sensors [9,10]. When the pointer on the LCD is located on the left end of the scale, the pointer in the computer is adjusted to the minimum position. When the pointer on the LCD is located on the middle of the scale, the computer pointer is adjusted to the middle. Based on the adjustments on these positions, the indicator with two laser displacement sensors can get the readings by computer automatically. The indicator with two laser displacement sensors used as a reading system of the mechanical balance is available for decreasing reading errors caused by human vision. Figure 6 shows the position of one laser displacement sensor and the software interface of the indicator.

### 2.3. Counter Weight Selecting System

**Figure 7:**
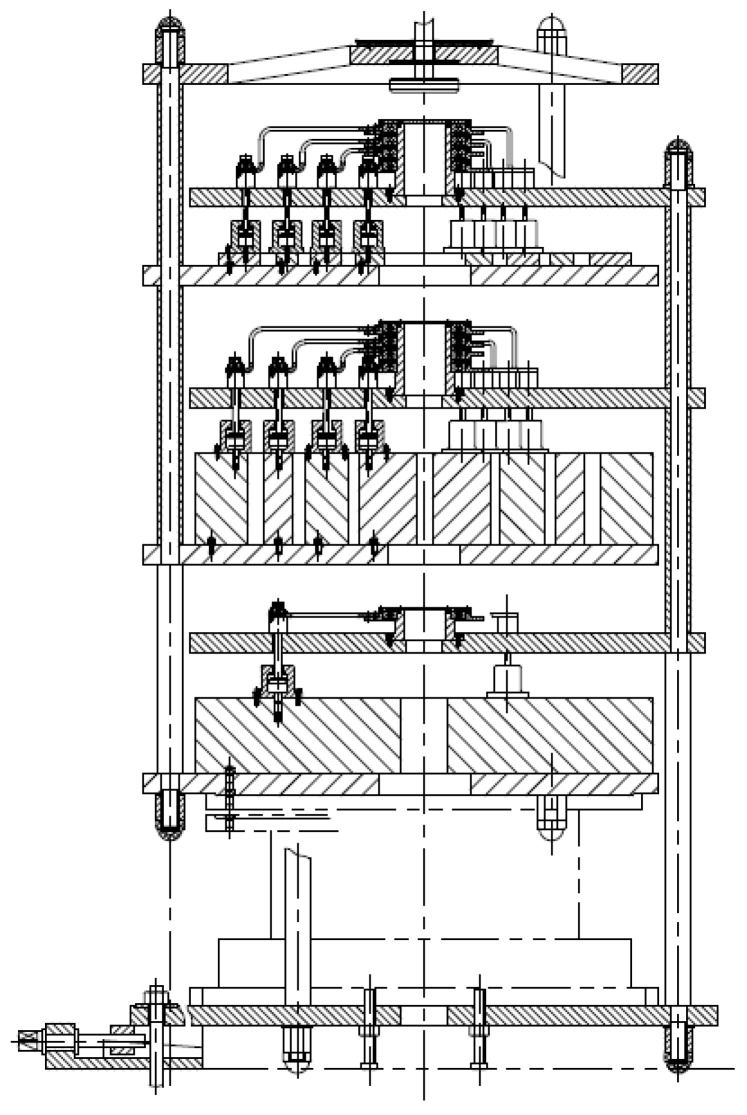
Counterweight selection system.

According to the measurement requirement of full range from 100 kg to 2000 kg in a limited space, a counterweight selection system is designed and produced. The counterweight selection system shown in Figure 7 consists of one stainless steel frame with three layer support, nested 1, 2, 2, 5 ratio counterweights from the inside to the outside and three sets of selection modules with nine motors for selecting the counterweights. The first selection module with four motors on the top of the counterweight selection system is used for selecting one weight or one set of weights among 10, 20, 20 and 50 kg options. The second one is similar as the first layer structure for selecting one weight or one set of weights among 100, 200, 200 and 500 kg options. There is one 1000 kg weight and one set of selection modules with one motor in the third layer.

### 2.4. Synchronous Lifting Control

To improve the repeatability and sensitivity of the mechanical balance, a synchronous lifting control is developed for lifting the weighing system and the counterweight selection system along the vertical direction synchronously [11]. Four motors under the weighing system and four motors under the counterweight selection system are driven synchronously for decreasing the displacement errors between them.

### 2.5. Monitor System for the Balance of the Beam

**Figure 8:**
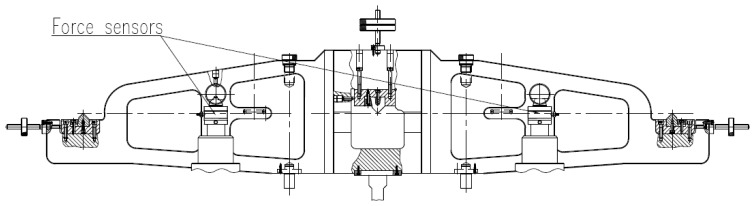
The force sensors installation positions.

After the weighing system and the counterweight selection system are lifted synchronously, the beam including the middle knife and side knives is dropped. The middle knife-edge and corresponding bearing block form the pivot to support everything. Four force sensors are mounted at two sides of the beam. The installation positions are shown in Figure 8. When the beam is dropping, the vertical forces of four points on the beam are measured to judge the loading synchronization and adjust the position of the beam.

## 3. Operational Principle

**Figure 9:**
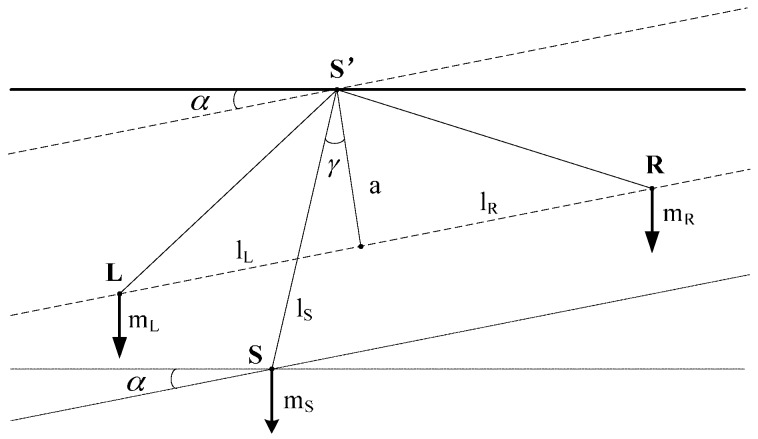
The general two-dimensional representation.

The weighing system is located at the left of the mechanical balance, and the counterweight selection system is on the right side. When a measurement is started, the reference weight and the test weight are place on the weighing system of the mechanical balance in turn. Meanwhile, according to the nominal mass of reference weight, a counterweight or a set of counterweights are selected on the counterweight selection system. Figure 9 shows a general two-dimensional representation of the mechanical balance showing the characteristic quantities of the mechanical balance. The point S’ is the beam's pivot. The gravity force of beam mass $m_{S}$ acts at its center of gravity S. The masses $m_{L}$ and $m_{R}$ hang at points L and R. The broken line connecting L and R forms an angle α with the horizon line through point S’, and is divided perpendicularly through S’ with the length a into the sections $l_{L}$ and $l_{R}$.

The lever arm of the balance's center of gravity with a length $l_{S}$ forms an angle γ with a. These torques around point S’ are neutralized under the equilibrium condition [12]. With the gravitational accelerations $g_{L}$, $g_{R}$ and $g_{S}$ in the three gravitational centers of the masses, the following expression is valid:

(1) $$m_{R}g_{R}\left(l_{L}\cos\alpha +a\sin\alpha \right)=m_{L}g_{L}\left(l_{L}\cos\alpha -a\sin\alpha \right)+m_{S}g_{S}l_{S}\sin\left(\gamma -\alpha \right)$$

It is assumed that $g_{L}=g_{R}=g_{S}=g$, the sensitivity of the mechanical balance is defined according to Equation (1) as follows:

(2) $$\frac{\partial \alpha }{\partial m_{L}}=\frac{l_{L}\cos\alpha -a\sin\alpha }{m_{L}\left(l_{L}\sin\alpha +a\cos\alpha \right)+m_{R}\left(a\cos\alpha -l_{R}\sin\alpha \right)+m_{S}l_{S}\cos\left(\gamma -\alpha \right)}$$

In the case of $a=0,\;l_{L}=l_{R}=l,\;\gamma =0\;\mathrm{and}\;\alpha =0$, Equation (1) is changed as follows:

(3) $$m_{L}=m_{R}=m$$

The sensitivity of mechanical balance is modified as follows:

(4) $$\frac{\partial \alpha }{\partial m_{L}}=\frac{l}{m_{S}l_{S}}$$

The sensitivity is independent of load, depending only on the mass of the beam and the position of the center of gravity. γ=0 means that the mechanical balance is symmetrical. The beam of a symmetrical balance is horizontal only if the masses of the ends of beam are equal. a=0,α=0 means that the pivots are on one level.

Based on the principle mentioned above, the production and assembly of one long beam with knives and bearing blocks is very important for improving the repeatability and sensitivity of a mechanical balance. Three coordinate measurements and adjusting technology is used for adjusting the straightness, parallelism and flatness between the middle knife and side knives to be mounted on the beam. The parallelism and the flatness between middle knife-edge and side knife-edges are less than 20 μm. The straightness of each knife-edge is also less than 20 μm.

## 4. Mechanical Balance Experiments

### 4.1. Mechanical Balance Results

Class E_2_ weights of 5, 10 and 20 g are used as sensitivity weights from no load to full load. Ten pieces of class E_2_ 50 kg weights are used as the reference weight of 500 kg. One hundred pieces of class F_1_ 20 kg weights are used as the reference weights for 1000 kg or 2000 kg. The steps of balance calibration are shown as follows:

(1) Under no loading conditions, one uses the small counterweights to adjust the balance of the beam.
(2) Record the indication of the balance.
(3) Add a sensitivity weight of 5 g on the left side.
(4) Record the indication of the balance.
(5) Remove the sensitivity weight of 5 g.
(6) Load the reference weights of 2000 kg on the left side and the 2000 kg counterweight on the right side.
(7) Increase or decrease the small counterweights until the beam is balanced.
(8) Record the indication of the balance.
(9) Add a sensitivity weight of 20 g on the left side.
(10) Record the indication of the balance.
(11) Remove the sensitivity weight of 20 g.
(12) Unload the reference weights of 2000 kg on both sides.
(13) Use the small counterweights to adjust the balance of the beam.
(14) Record the indication of the balance.
(15) Add a sensitivity weight of 5 g on the right side.
(16) Record the indication of the balance.
(17) Remove the sensitivity weight of 5 g.
(18) Repeat (6) and (7).
(19) Record the indication of the balance.
(20) Add a sensitivity weight of 20 g on the right side.
(21) Record the indication of the balance.

The sensitivities of no load and full load are shown as Table 1. The full load sensitivity is more than 1.7 part in 10^−4^ rad/g. The standard deviations of no load and full load are shown in Table 2 and Table 3, respectively.

**Figure 10:**
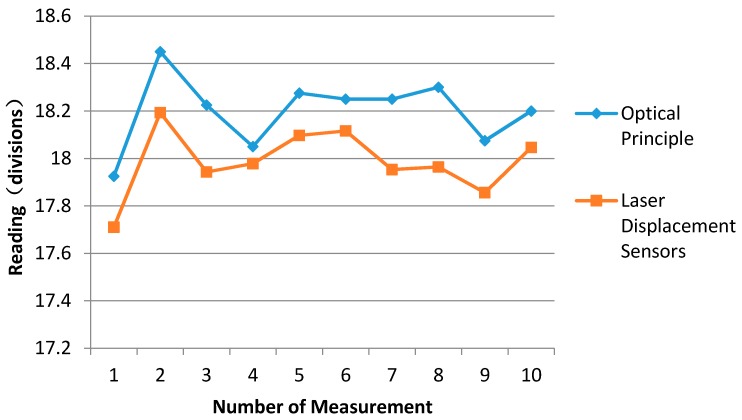
Reading results of two indicators.

After the indicator based on laser displacement sensors is adjusted, the reading comparisons between indicators based on optical principles and laser displacement sensors at 500 kg are shown as Table 4 and Figure 10. The comparison steps between indicators are as follows:

(1) Place the 500 kg weight on the weighing pan (left side of the mechanical balance) and select a 500 kg counterweight on the right side of the mechanical balance.
(2) Read the first divisions from the two indicators, respectively, after reaching balance.
(3) Place a sensitivity weight of 5 g on the top of the counterweights or the weighing pan.
(4) Read the second divisions, respectively.
(5) Divide 5 g into the difference between the first divisions and the second divisions. The mass against one division that represents the sensitivity of the mechanical balance at 500 kg could be known.

The values of the first column in the Table 4 are the readings based on the optical principle, and the values of the second column are the sensitivities corresponding to the readings. The values of the third column in the Table 4 are the readings based on two laser displacement sensors. The values of the last column are the sensitivities corresponding to the readings based on two laser displacement sensors. The maximum difference between readings based on optical principle and two laser displacement sensors is from the eighth row. It is 0.336 divisions. The mass corresponding to the divisions is 0.2 g, i.e., very small and it can be ignored. The results show the readings between two indicators coincide.

**Figure 11:**
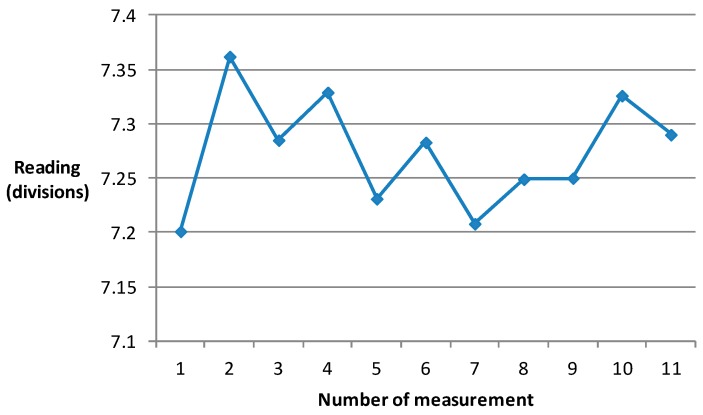
Measurement of beam stability.

The results regarding the measurement of beam stability at 2000 kg are shown in Figure 11. The maximum value is 7.201 divisions, and the minimum value is 7.329 divisions. The difference between the maximum and minimum values is only 0.118 divisions.

**Figure 12:**
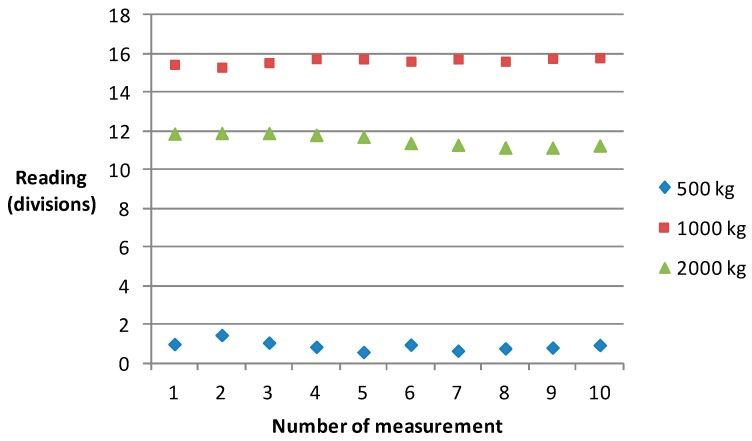
The results for 500 kg, 1000 kg and 2000 kg.

**Figure 13:**
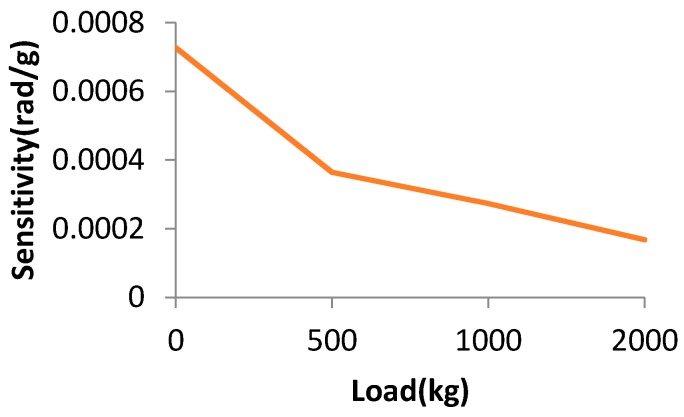
The relationship between the sensitivity and the load.

Figure 12 shows the results of 500, 1000 and 2000 kg. The sensitivity for 500 kg is more than 3.6 parts in 10^−4^ rad/g, the sensitivity for 1000 kg is more than 2.7 parts in 10^−4^ rad/g, and the sensitivity for 2000 kg is more than 1.7 parts in 10^−4^ rad/g. Figure 13 shows the relationship between the sensitivity and the load. As the load changes, the sensitivity becomes worse.

### 4.2. Uncertainties of Mechanical Balance

According to the operational principle of the mechanical balance, the expression of indication error with respect to the mechanical balance is as follows:

(5) E=I−L

where *E* is the error between the indication of the mechanical balance and the applied load, *I* is the indication of mechanical balance regarding applied load and *L* is the applied load [13,14,15].

The uncertainty of the mechanical balance is as follows:

(6) $$u^{2}\left(E\right)=s^{2}\left(I\right)+\frac{d_{0}^{2}}{12}+\frac{d_{L}^{2}}{12}+u^{2}\left(L\right)$$

where u(E) is the uncertainty of error, s(I) is the repeatability of the mechanical balance corresponding to the applied load, $d_{0}$ is the no load sensitivity, $d_{L}$ is the applied load sensitivity, u(L) is the uncertainty deriving from the reference weight including the air buoyancy correction. The uncertainties of 500, 1000 and 2000 kg are shown in Table 5.

## 5. Conclusions

The establishment of a new 2000 kg mechanical balance is important for traceability of class F_1_ weights from 500 kg to 2000 kg used in industrial processes. To develop a mechanical balance with high accuracy and a wide measurement range for measurement of class F_1_ weights over 500 kg, a new heat treatment method of metal material, three coordinate measurement and adjustment technology, dynamic monitoring with regards to the balance of the beam and synchronous lifting control are used to improve the repeatability and sensitivity of the mechanical balance. For the realization of any combinations of counterweights in a limited space, a new counter-weight selection system is designed and its construction accomplished. The sensitivity of the new 2000 kg mechanical balance is more than 1.7 parts in 10^−4^ rad/g. The extended uncertainties of 500, 1000 and 2000 kg for the mechanical balance are 0.47 g, 1.8 g and 3.5 g, respectively. The results show the performance of the new mechanical balance is better than that of electronic mass comparators used in commerce.

## Figures and Tables

**Table 1:** Sensitivity measurements of the mechanical balance.

Left Side	Right Side	Results (Division)	Sensitivity (10^−4^ rad/g)
No load	No load	15.381	7.3
Sensitivity weight 5 g	No load	32.072	
Full load	Full load	18.694	1.7
Sensitivity weight 20 g	Full load	34.130	
No load	No load	15.803	7.3
No load	Sensitivity weight 5 g	−4.177	
Full load	Full load	18.838	1.7
Full load	Sensitivity weight 20 g	3.298	

**Table 2:** Standard deviation of no load.

Results (Division)	Sensitivity (10^−4^ rad/g)	Standard Deviation (g)
15.394	7.3	0.04
15.515	7.3	
15.637	7.3	
15.714	7.3	
15.452	7.3	
15.546	7.3	

**Table 3:** Standard deviation of full load.

Results (Division)	Sensitivity (10^−4^ rad/g)	Standard Deviation (g)
18.765	1.7	0.13
18.783	1.7	
18.888	1.7	
19.026	1.7	
18.896	1.7	
18.954	1.7	

**Table 4:** Reading comparisons between indicators based on optical principle and laser displacement sensors at 500 kg.

Optical Principle		Laser Displacement Sensors	
Results (Division)	Sensitivity (10^−4^ rad/g)	Results (Division)	Sensitivity (10^−4^ rad/g)
17.925	3.75	17.710	3.73
18.450	3.69	18.193	3.60
18.225	3.64	17.943	3.55
18.050	3.54	17.978	3.50
18.275	3.61	18.097	3.54
18.250	3.63	18.116	3.57
18.250	3.64	17.953	3.55
18.300	3.62	17.964	3.54
18.075	3.76	17.856	3.74
18.200	3.65	18.046	3.58

**Table 5:** The extended uncertainties of 500, 1000 and 2000 kg.

Load (kg)	Standard Uncertainty of the Weighing Process (g)	Standard Uncertainty Due to the Sensitivity (g)	Standard Uncertainty of the Reference Weight with Air Buoyancy Correction (g)	Combined Standard Uncertainty (g)	Extended Uncertainty (k = 2, g)
500	0.072	0.176	0.134	0.232	0.47
1000	0.028	0.236	0.833	0.867	1.8
2000	0.117	0.469	1.667	1.736	3.5
